# Supplementary material for: Decoding pH‐Driven Phase Transition of Lipid Nanoparticles
Source: Small. 2026 Jan 17;22(14):e11381. doi: 10.1002/smll.202511381 (PMC12965128; doi:10.1002/smll.202511381)
Supplement: Supplementary file 1 — Supporting File: smll72336‐sup‐0001‐SuppMat.pdf. [file SMLL-22-e11381-s001.pdf]

Supporting Information:

Decoding pH-Driven Phase Transition of Lipid Nanoparticles

*Marius F.W. Trollmann Rainer A. Böckmann**

Marius F.W. Trollmann

Computational Biology, Department of Biology, Friedrich-Alexander-Universität Erlangen-Nürnberg, Erlangen, Germany

Erlangen National High-Perfomance Computing Center (NHR@FAU), Erlangen, Germany

Email Address: marius.trollmann@fau.de

Prof. Dr. Rainer A. Böckmann

Computational Biology, Department of Biology, Friedrich-Alexander-Universität Erlangen-Nürnberg, Erlangen, Germany

Erlangen National High-Perfomance Computing Center (NHR@FAU), Erlangen, Germany

FAU Research Center New Bioactive Compounds (FAU NeW), Erlangen, Germany

FAU Profile Center Immunomedicine (FAU I-MED), Erlangen, Germany

Email Address: rainer.boeckmann@fau.de

The PDF file includes

- Supporting Figures S1 – S26,
- Supporting References

Supplementary Figures

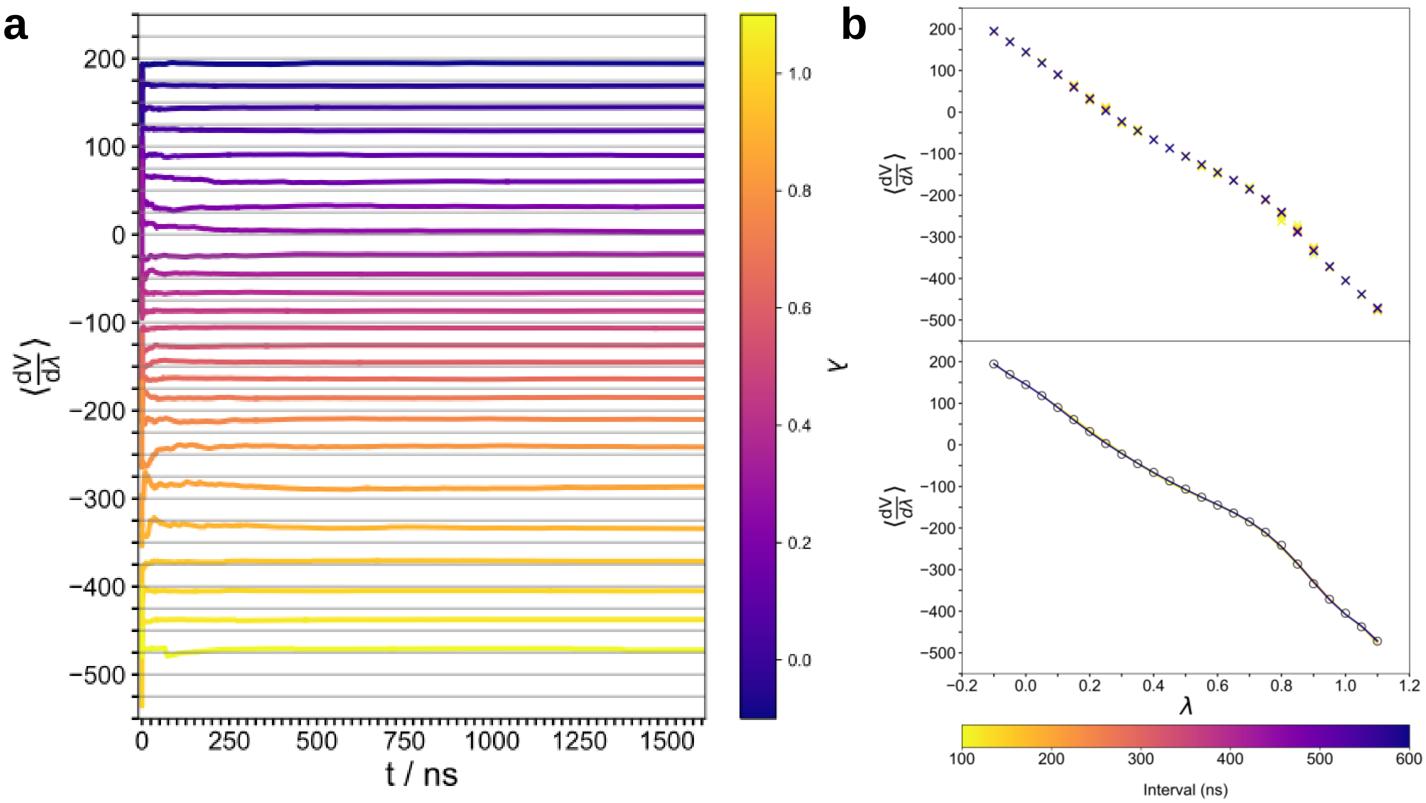

Figure S1: **a** Cumulative estimation of $\langle \frac{dV}{d\lambda} \rangle$ as a function of increasing simulation length. **b** Cumulative estimation of $\langle \frac{dV}{d\lambda} \rangle$ as a function of λ (upper panel), and the development of the 9th-order polynomial fit for $\langle \frac{dV}{d\lambda} \rangle$ under an increasing simulation length (bottom panel). The black circles in the bottom panel represent the final estimate for $\langle \frac{dV}{d\lambda} \rangle$ using the whole equilibrated range of the trajectory from 100 ns to 1700 ns.

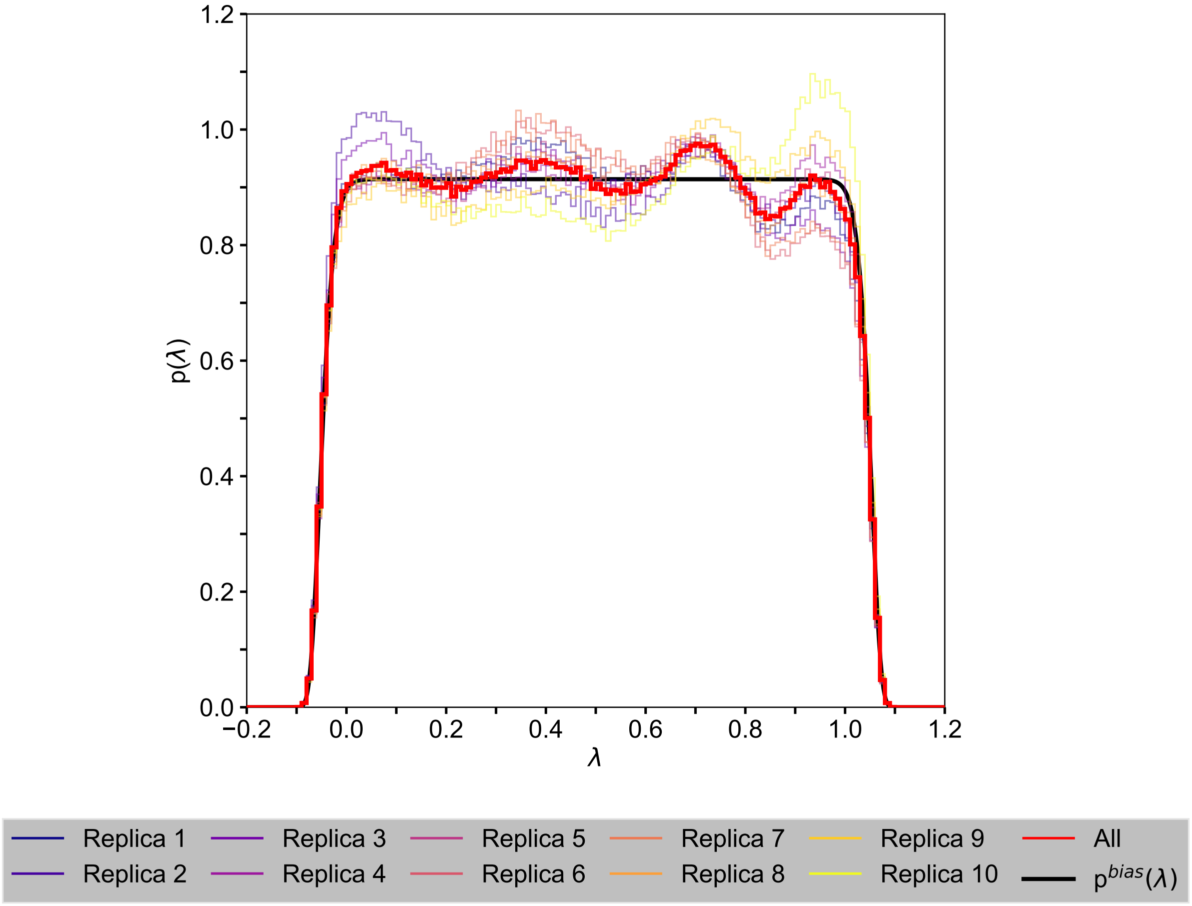

Figure S2: Distribution of the λ-coordinate for the aminolipid obtained from ten independent simulations applying only the correction potential $V^{MM}(\lambda)$ for the protonation free energy and the $V^{\mathrm{bias}}(\lambda)$ (barrier height set to 0.0 kJ mol^{-1}). The expected probability density function, $p^{\mathrm{bias}}(\lambda)$, shown for reference, is derived from the applied bias potential, $V^{\mathrm{bias}}(\lambda)$.

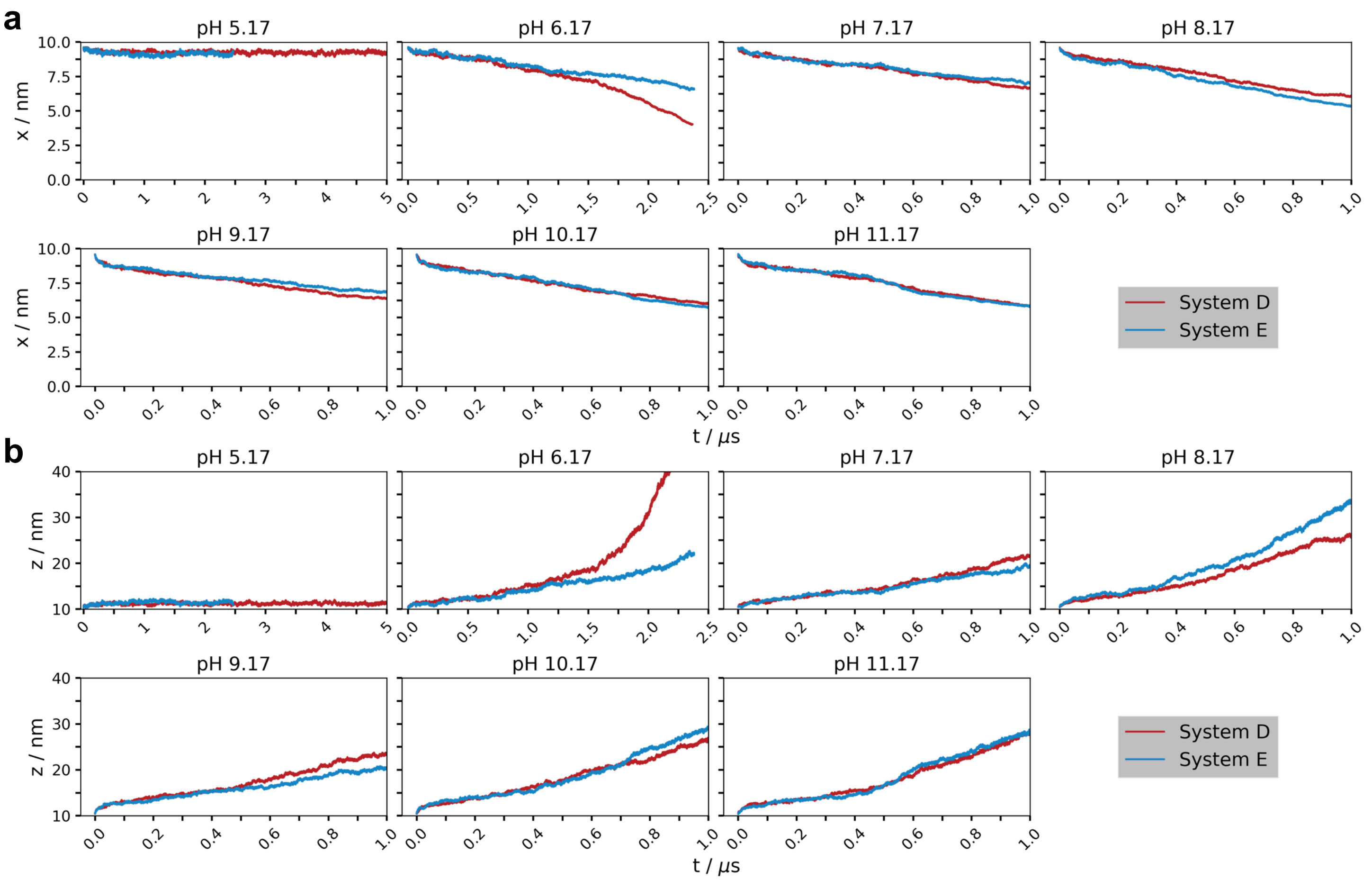

Figure S3: Length of the box vectors in **a** the x-direction and **b** the z-direction over time for lipid mixture simulations using either $2 \cdot N + 1$ buffer particles for N titratable sites (System D, Tab. 1) or half that number (System E, Tab. 1).

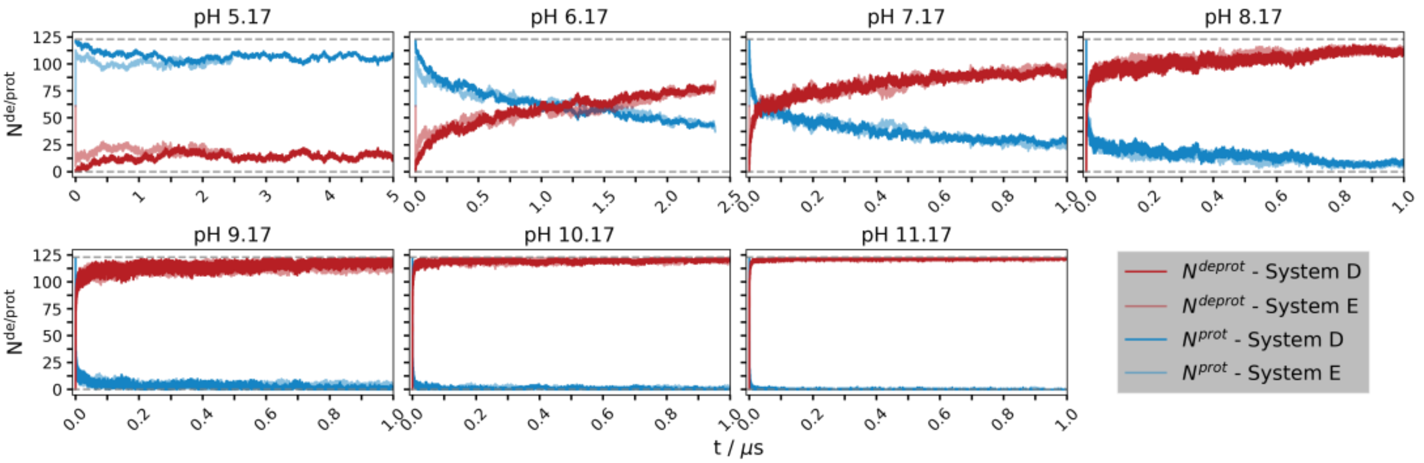

Figure S4: Number of (de)protonated residues in lipid mixture simulations using either $2 \cdot N + 1$ buffer particles for N titratable sites (System D, Tab. 1) or half that number (System E, Tab. 1). The number of (de)protonated residues was calculated every 1 ps based on the λ-coordinate for each aminolipid ($\lambda < 0.2$, protonated; $\lambda > 0.8$, deprotonated).

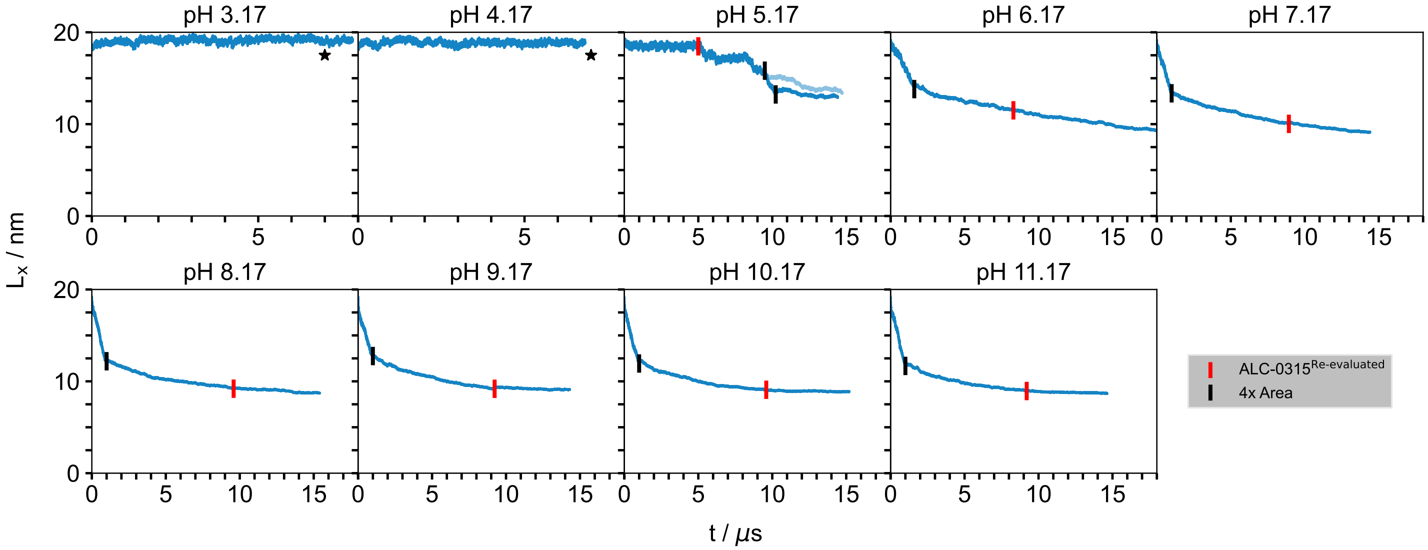

Figure S5: Length of the box vector in the x-direction over time for simulations of systems $D_{pH\ 5.17}$, F^{1-6}, $D^{\ddagger}$, and $F^{0-6\ddagger}$(see Tab. 1). Black vertical lines indicate the quadrupling of the membrane surface, performed to prevent periodic boundary artifacts caused by an undersized surface area, while red vertical lines indicate the restart of the simulations with corrected polynomial coefficients (ALC-0315$^{\text{Re-evaluated}}$). Box lengths prior to the black vertical bar, or annotated with a star ($\star$), were doubled to produce a continuous curve in the plot.

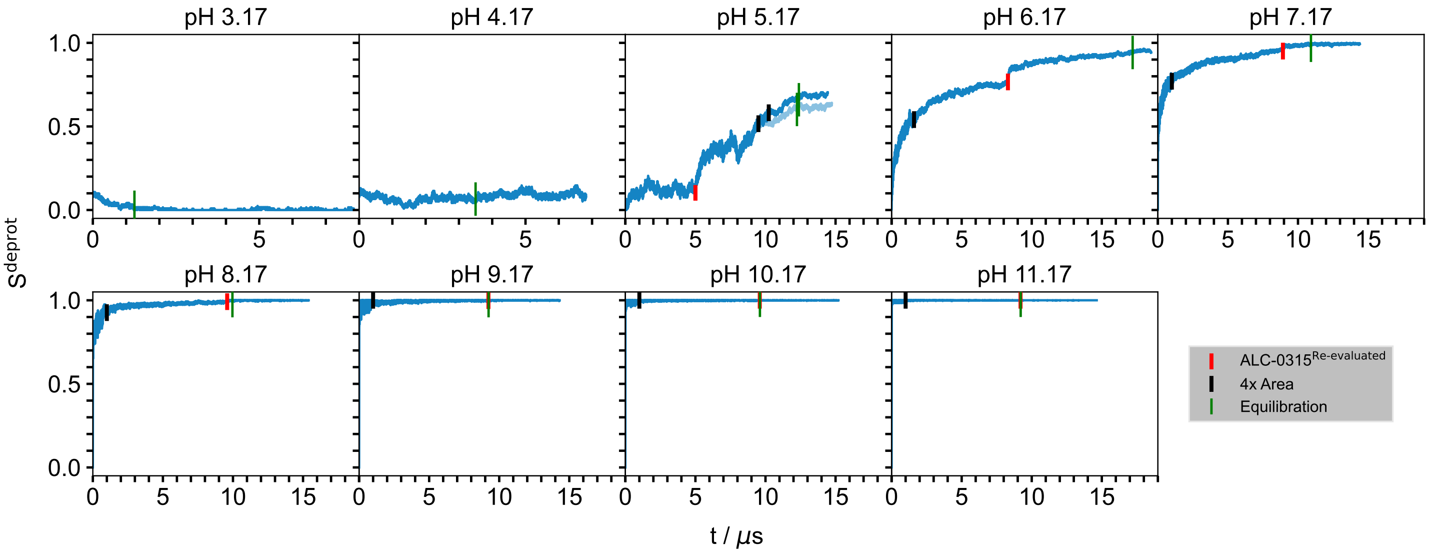

Figure S6: Fraction of deprotonation (S^{deprot}) (see Eq. 3) over time is shown for simulations of $D_{pH\ 5.17}$, F^{1-6}, $D^{\ddagger}$, and $F^{0-6\ddagger}$ (see Tab. 1). The number of (de)protonated aminolipids was obtained via the λ-coordinate of each aminolipid ($\lambda < 0.2$, protonated; $\lambda > 0.8$, deprotonated) every 1 ps. Black vertical lines indicate the quadrupling of the membrane surface, performed to prevent periodic boundary artifacts caused by an undersized surface area, red vertical lines indicate the restart of the simulations with corrected polynomial coefficients (ALC-0315$^{\text{Re-evaluated}}$), and green vertical lines indicate the convergence of S^{deprot}.

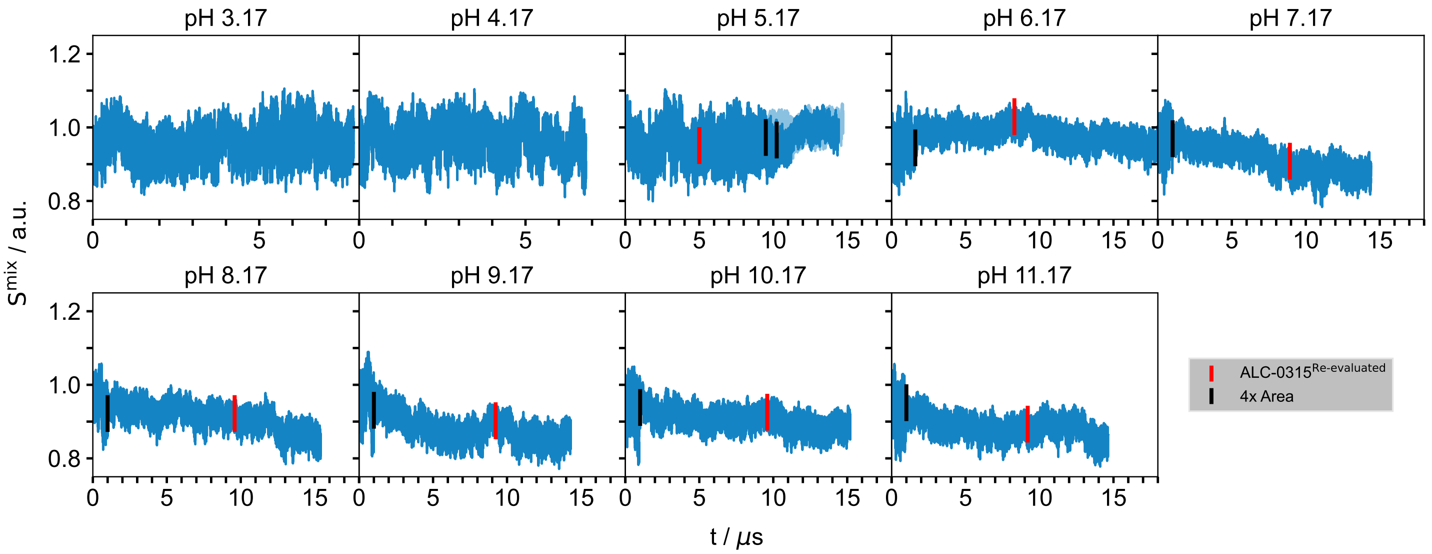

Figure S7: Conditional mixing entropy (S^{mix}) over time is shown for simulations of $D_{\text{pH 5.17}}$, F^{1-6}, $D^{\ddagger}$, and $F^{0-6\ddagger}$(see Tab. 1). S^{mix} was calculated following the method of Brandani *et al.*,[1] using a three-dimensional Euclidean distance cutoff of 1 nm to identify neighboring lipids. Lower values correspond to a stronger demixing of the lipid components. Black vertical lines indicate the quadrupling of the membrane surface, performed to prevent periodic boundary artifacts caused by an undersized surface area, while red vertical lines indicate the restart of the simulations with corrected polynomial coefficients (ALC-0315$^{\text{Re-evaluated}}$).

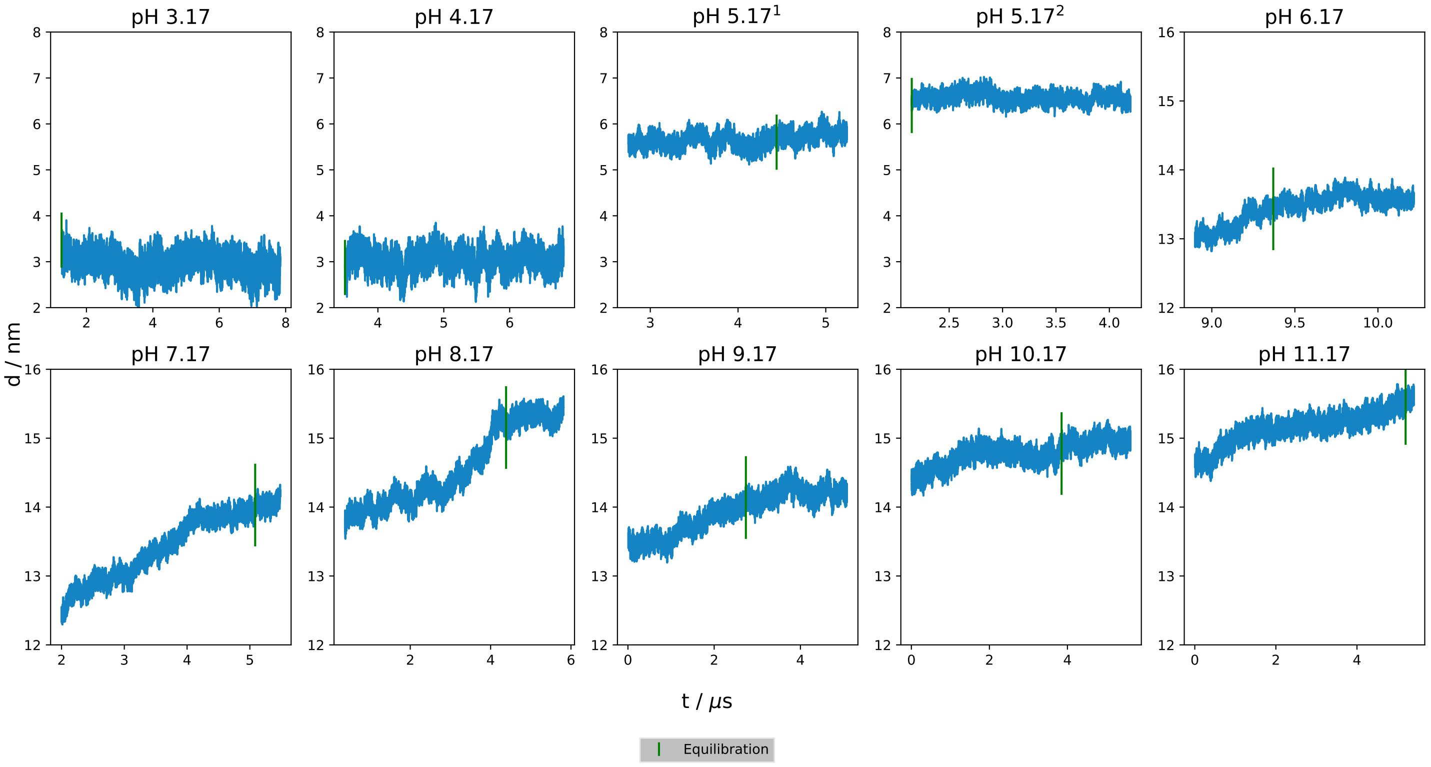

Figure S8: Membrane thickness as a function of time is shown for simulations of $F^{0\text{-}6\ddagger}$(see Tab. 1). The thickness was defined as the distance between the median z-positions of the phosphorus atoms of DSPC in the two leaflets. The curves do not start at 0 ns because only trajectory segments after equilibration of S^{deprot} were considered for calculating the membrane thickness. Green vertical lines indicate the convergence of the observable.

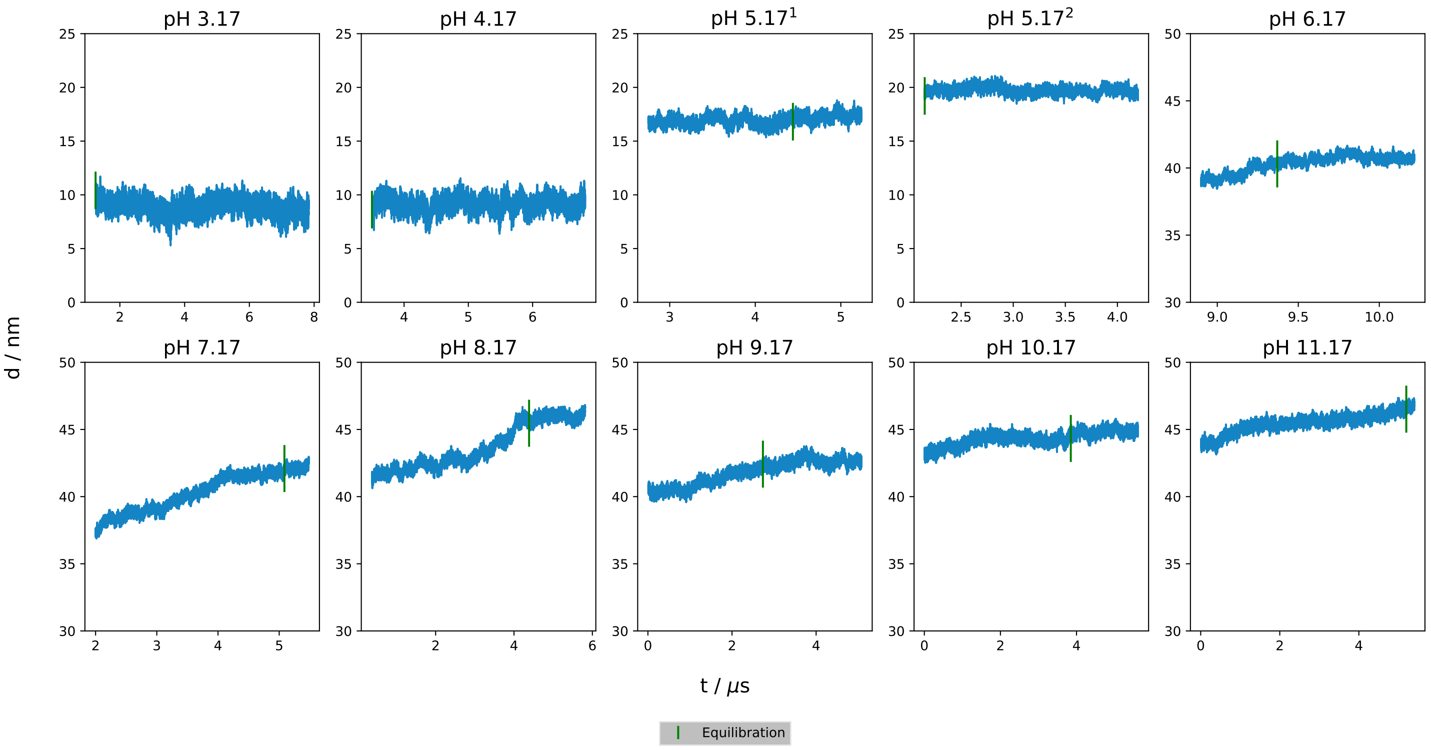

Figure S9: Estimated LNP diameter as a function of time is shown for simulations of $F^{0\text{-}6\ddagger}$(see Tab. 1). The diameter was obtained from the surface-to-volume ratio of the LNP-mimetic systems. The curves do not start at 0 ns because only trajectory segments after equilibration of S^{deprot} were considered for calculating the LNP diameter. Green vertical lines indicate the convergence of the observable.

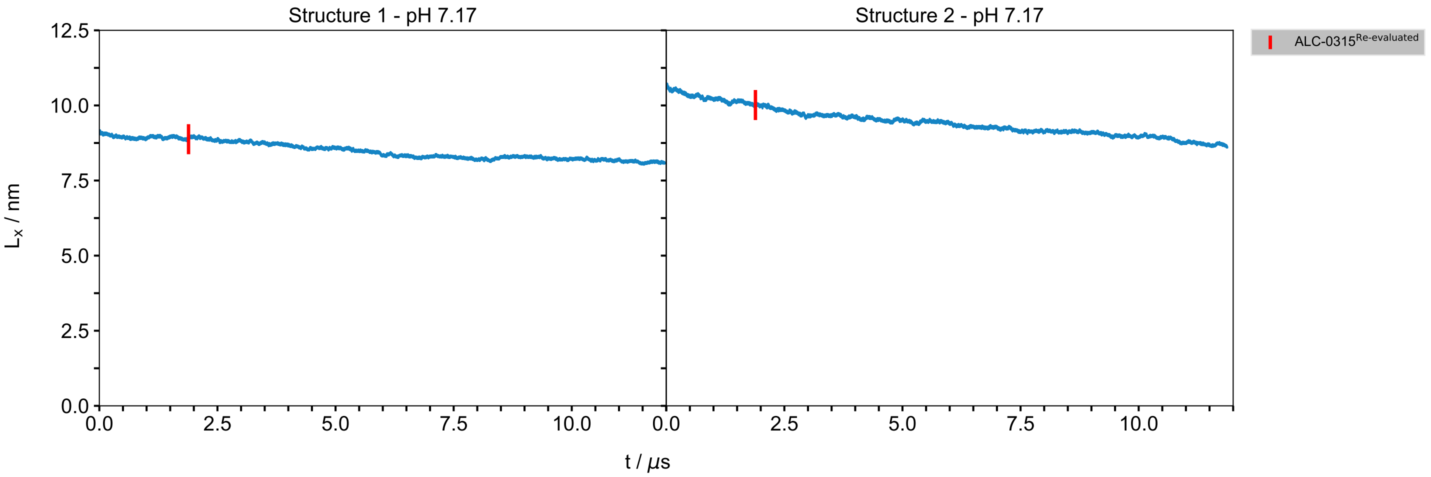

Figure S10: Length of the box vector in the x-direction over time for simulations of systems G and $G^{\ddagger}$ (see Tab. 1; LNP mixture containing mRNA). Red vertical lines indicate the restart of the simulations with corrected polynomial coefficients ($\text{ALC-0315}^{\text{Re-evaluated}}$).

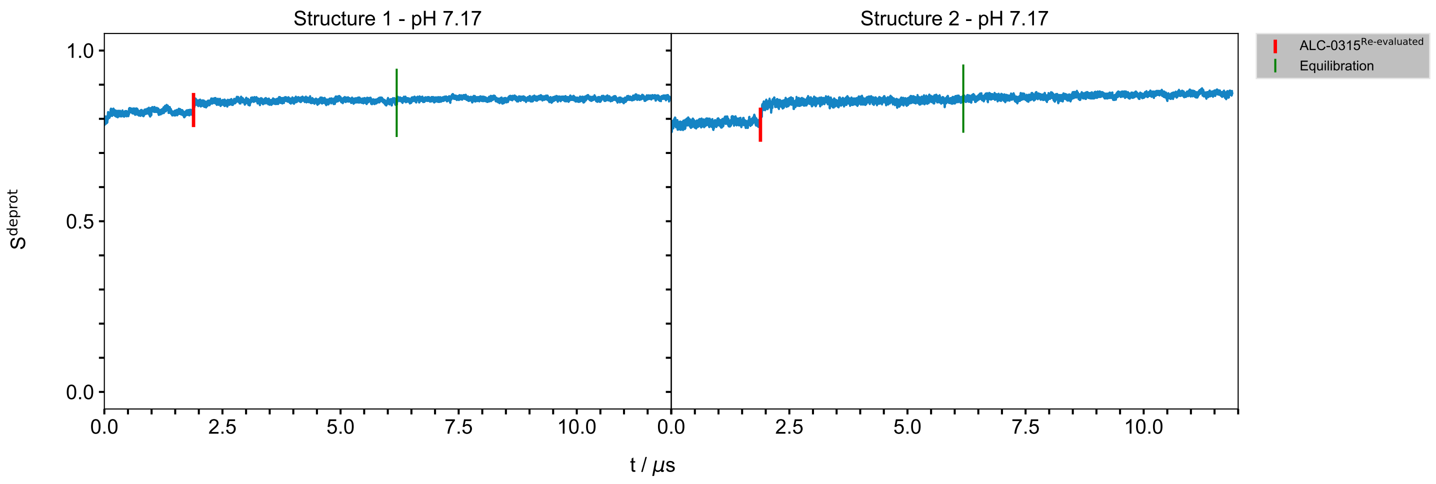

Figure S11: Fraction of deprotonation (S^{deprot}) (see Eq. 3) over time is shown for simulations of systems G and G‡ (see Tab. 1; LNP mixture containing mRNA). The number of (de)protonated aminolipids was obtained via the λ-coordinate of each aminolipid ($\lambda < 0.2$, protonated; $\lambda > 0.8$, deprotonated) every 1 ps. Red vertical lines indicate the restart of the simulations with corrected polynomial coefficients (ALC-0315$^{\text{Re-evaluated}}$), and green vertical lines indicate the convergence of S^{deprot}.

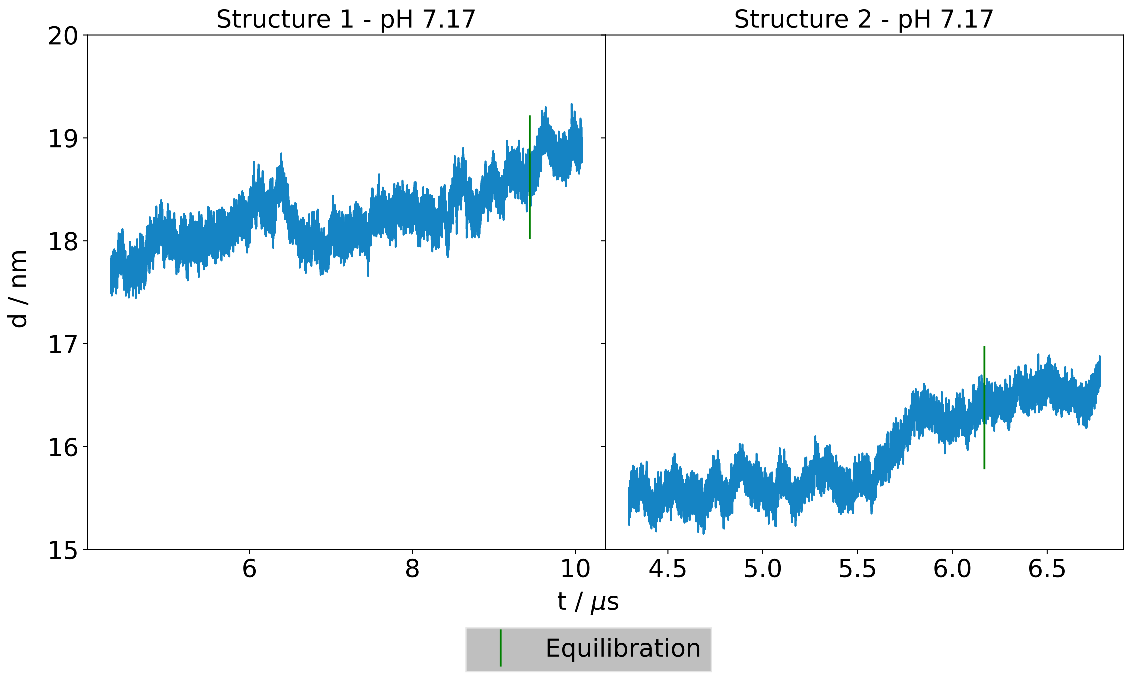

Figure S12: Membrane thickness as a function of time is shown for simulations of G‡ (see Tab. 1; LNP mixture containing mRNA). The thickness was defined as the distance between the median z-positions of the phosphorus atoms of DSPC in the two leaflets. The curves do not start at 0 ns because only trajectory segments after equilibration of S^{deprot} were considered for calculating the membrane thickness. Green vertical lines indicate the convergence of the observable.

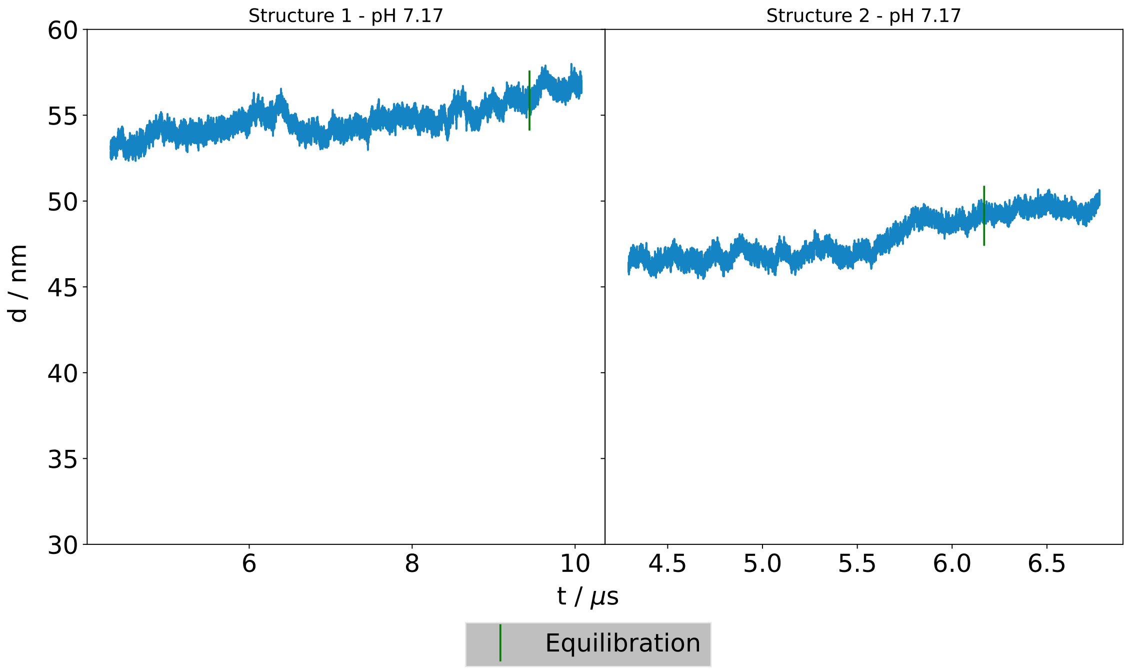

Figure S13: Estimated LNP diameter as a function of time is shown for simulations of $G^{\ddagger}$ (see Tab. 1; LNP mixture containing mRNA). The diameter was obtained from the surface-to-volume ratio of the LNP-mimetic systems. The curves do not start at 0 ns because only trajectory segments after equilibration of S^{deprot} were considered for calculating the LNP diameter. Green vertical lines indicate the convergence of the observable.

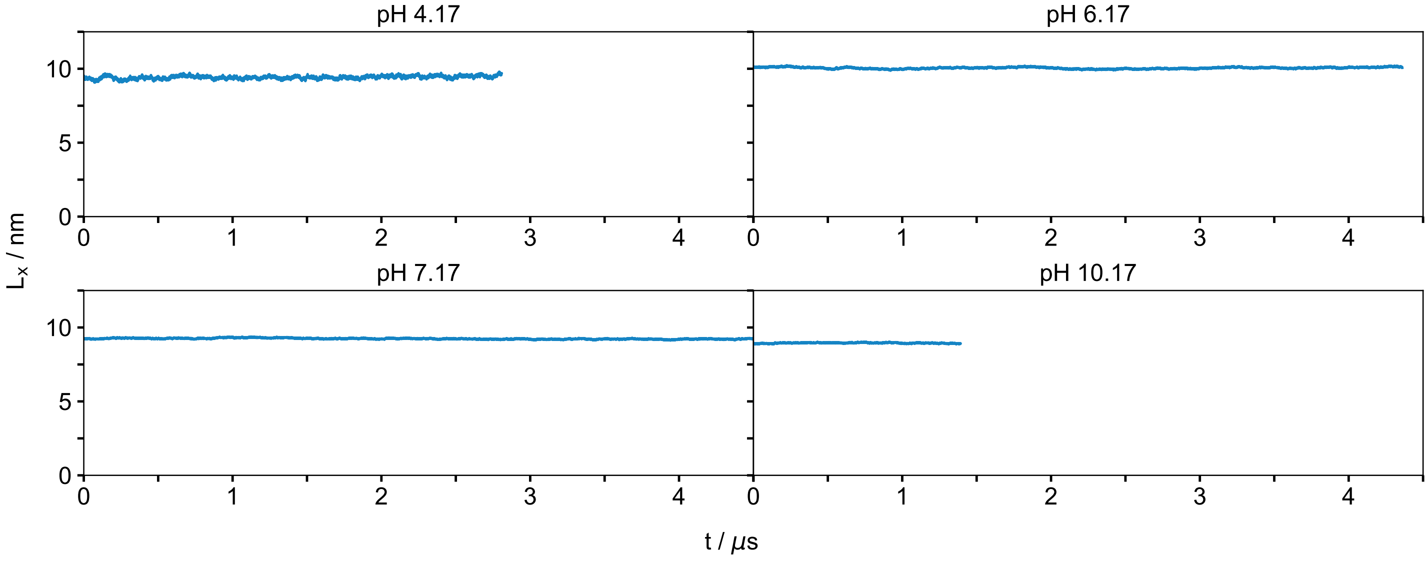

Figure S14: Length of the box vector in the x-direction over time for simulations of systems $H^{0-3\ddagger}$ (see Tab. 1; LNP mixture containing TNS).

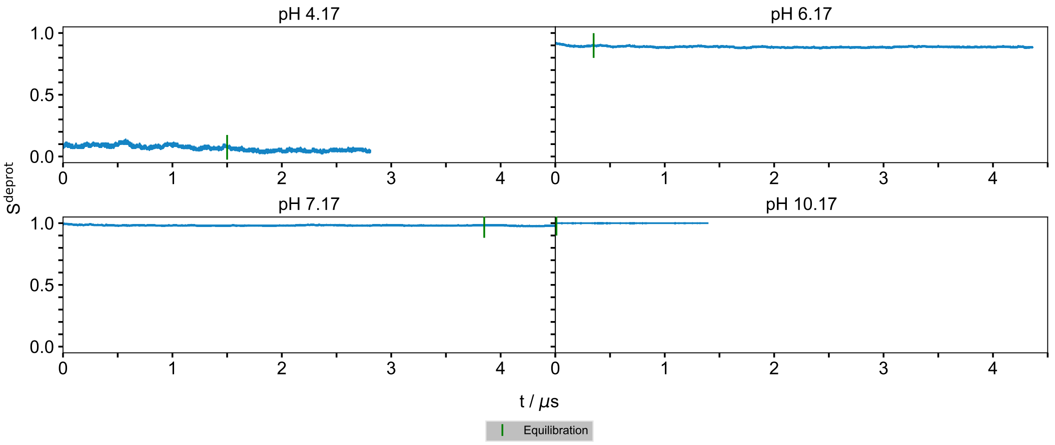

Figure S15: Fraction of deprotonation (S^{deprot}) (see Eq. 3) over time is shown for simulations of $H^{0-3\ddagger}$ (see Tab. 1; LNP mixture containing TNS). The number of (de)protonated aminolipids was obtained via the λ-coordinate of each aminolipid ($\lambda < 0.2$, protonated; $\lambda > 0.8$, deprotonated) every 1 ps. Green vertical lines indicate the convergence of S^{deprot}.

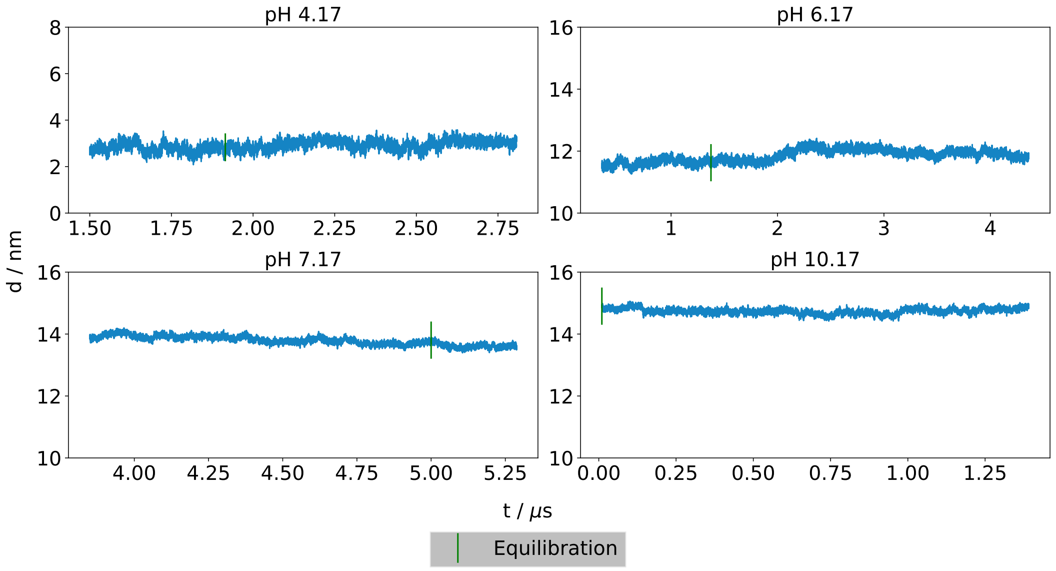

Figure S16: Membrane thickness as a function of time is shown for simulations of $H^{0-3\ddagger}$ (see Tab. 1; LNP mixture containing TNS). The thickness was defined as the distance between the median z-positions of the phosphorus atoms of DSPC in the two leaflets. The curves do not start at 0 ns because only trajectory segments after equilibration of S^{deprot} were considered for calculating the membrane thickness. Green vertical lines indicate the convergence of the observable.

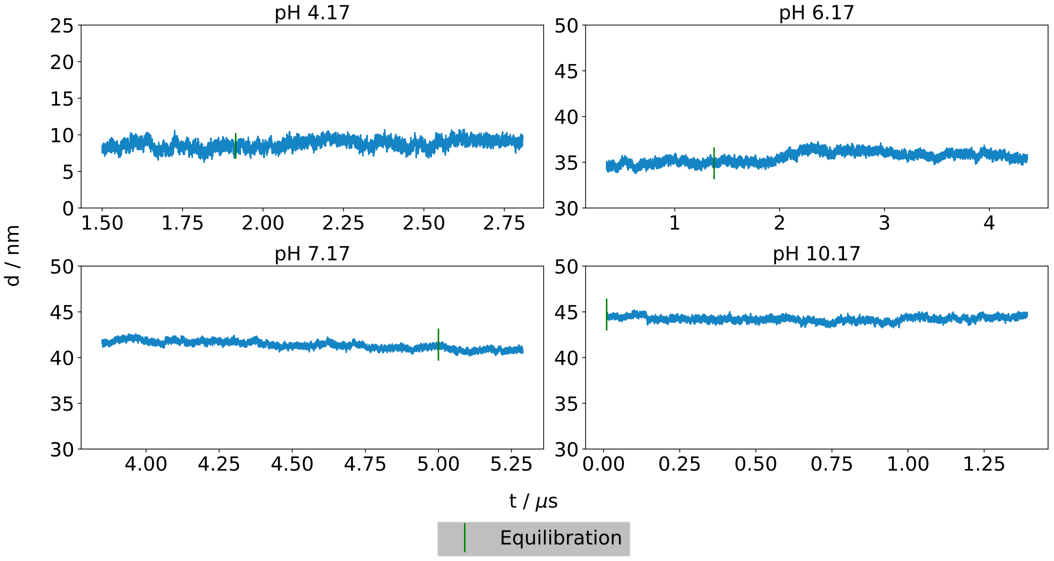

Figure S17: Estimated LNP diameter as a function of time is shown for simulations of $H^{0-3‡}$ (see Tab. 1; LNP mixture containing TNS). The diameter was obtained from the surface-to-volume ratio of the LNP-mimetic systems. The curves do not start at 0 ns because only trajectory segments after equilibration of S^{deprot} were considered for calculating the LNP diameter. Green vertical lines indicate the convergence of the observable.

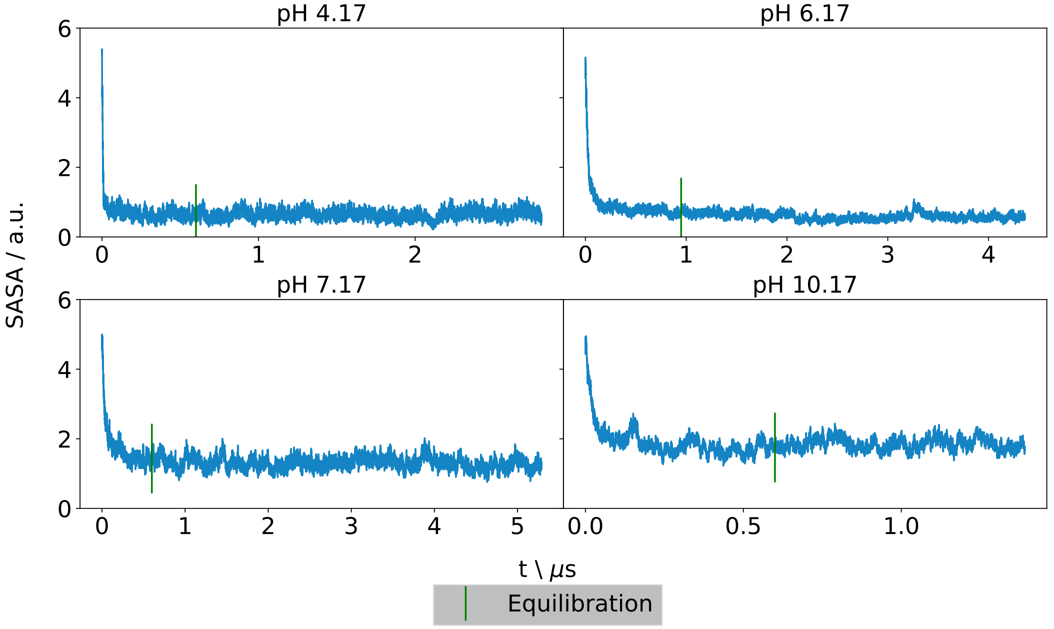

Figure S18: The solvent-accessible surface area (SASA) of TNS, averaged over all molecules, is shown as a function of time for simulations of $H^{0\text{-}3‡}$ (see Tab. 1; LNP mixture containing TNS). SASA was calculated using *gmx sasa* with the default probe radius of 0.14 nm. Green vertical lines indicate the convergence of the observable.

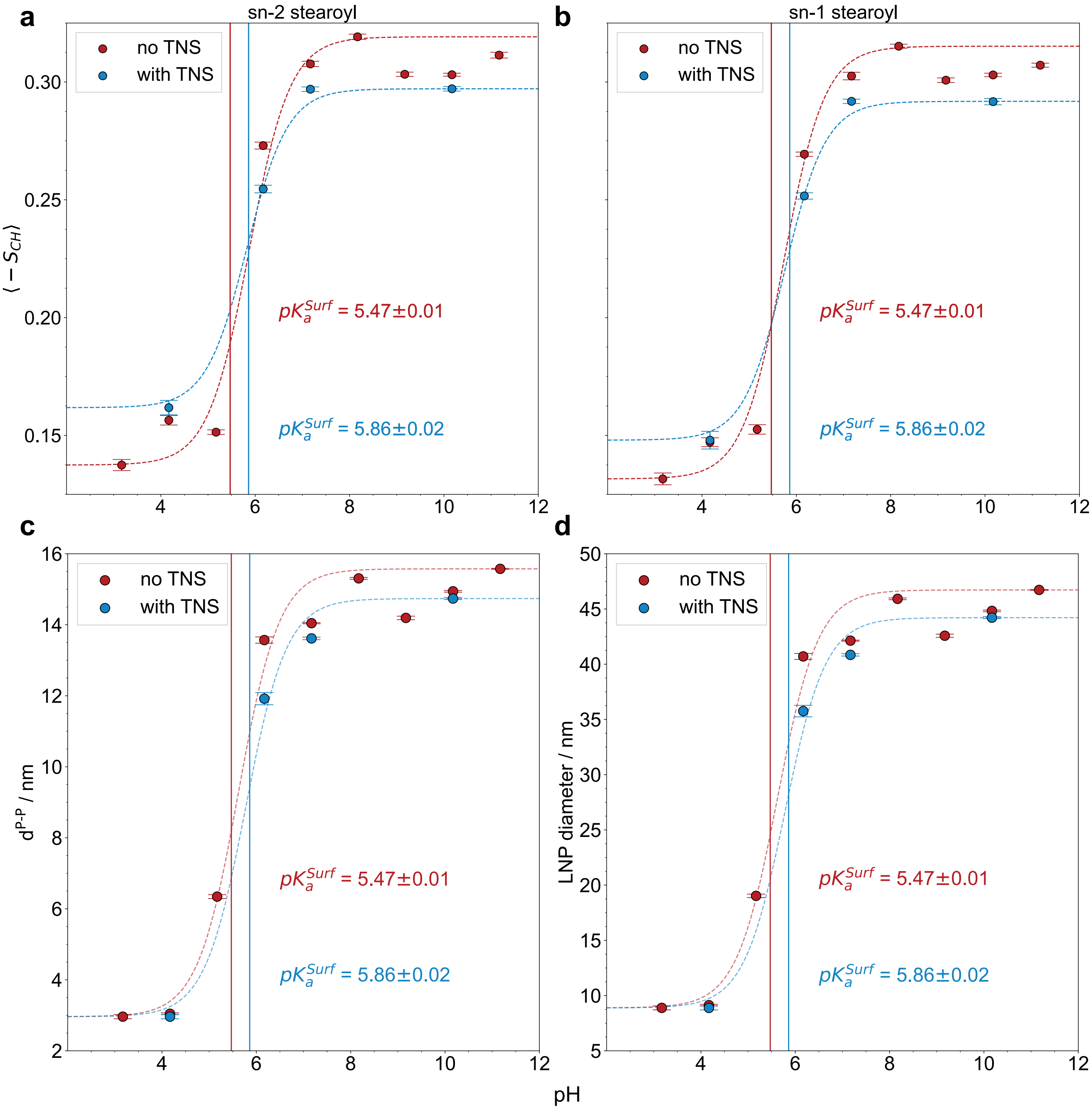

Figure S19: Mean deuterium order parameters for the **a** sn-2 stearoyl and **b** sn-1 stearoyl acyl chains, averaged over all DSPC molecules, as a function of pH. Deuterium order parameters were calculated using gorder.[2] **c** Membrane thickness of the LNP-mimetic systems, defined as the distance along the membrane normal (z-axis) between the median positions of the DSPC phosphate groups in each leaflet. **d** Effective LNP diameter estimated by assuming a constant volume-to-area ratio between the periodic LNP-mimetic systems and spherical LNPs. Values for systems at pH < 5 are shown for completeness but should be interpreted with caution, as these systems lack a core–shell organization rendering the constant volume-to-area ratio assumption invalid (at low pH, vesicular rather than core-shell LNP structures are expected). All quantities are shown for mRNA-free LNP-mimetic systems with and without TNS. Lines are guides to the eye. Error bars for the pK_a values represent 95% confidence intervals from bootstrapping, whereas all other error bars correspond to the standard error of the mean estimated by block averaging.[3] For the deuterium order parameters, the block-averaging implementation in *gorder* was used with a total of five blocks.

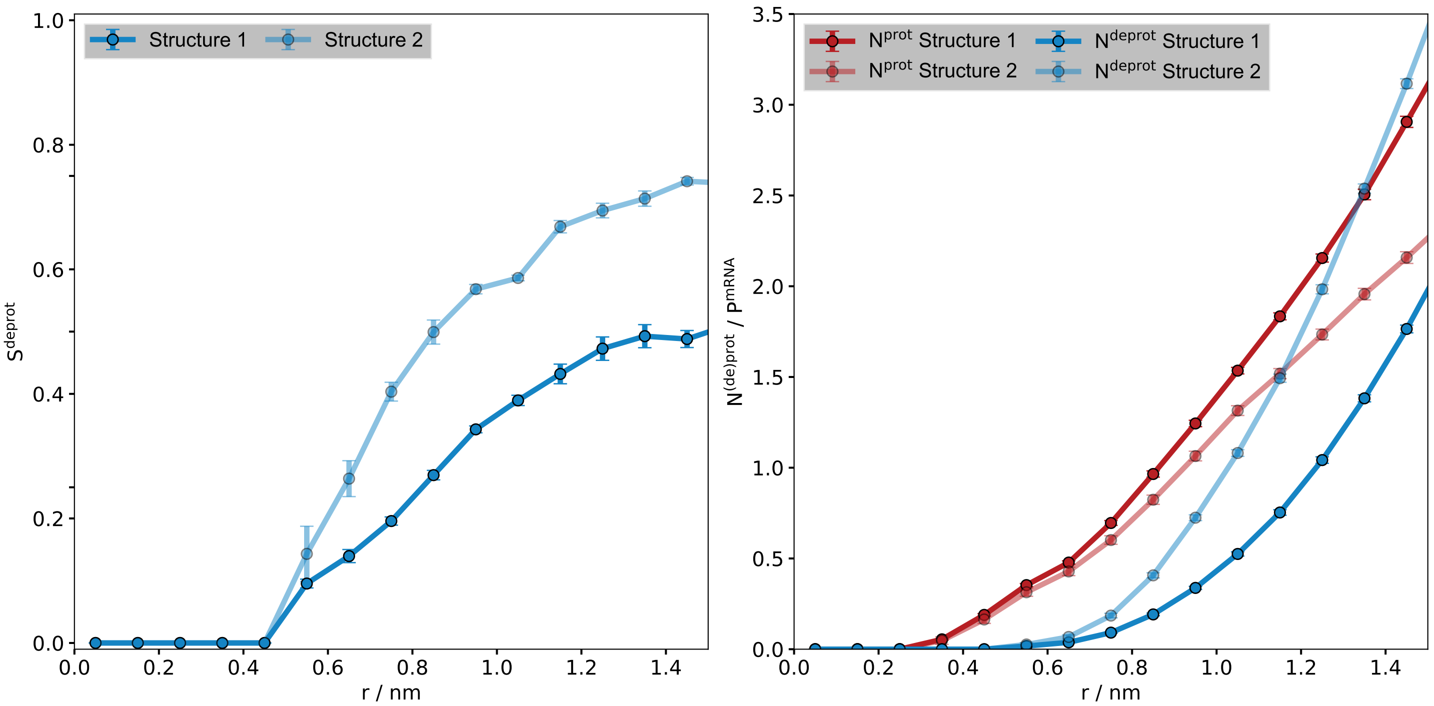

Figure S20: **a** Fraction of deprotonated ALC-0315 (S^{deprot}), **b** cumulative number of protonated ALC-0315 ($\lambda < 0.2$), and **c** cumulative number of deprotonated ALC-0315 ($\lambda > 0.8$) around the negatively charged backbone of the mRNA strands (see system G‡, Tab. 1). Note that the cumulative numbers are normalized by the amount of negatively charges phosphates in the mRNA backbone (here, $n^{PO_4^-}$=76). Distances were calculated between the nitrogen atom of ALC-0315 and the phosphorus atom in the nucleotide backbone. Averages were calculated after the S^{deprot} values reached equilibrium.

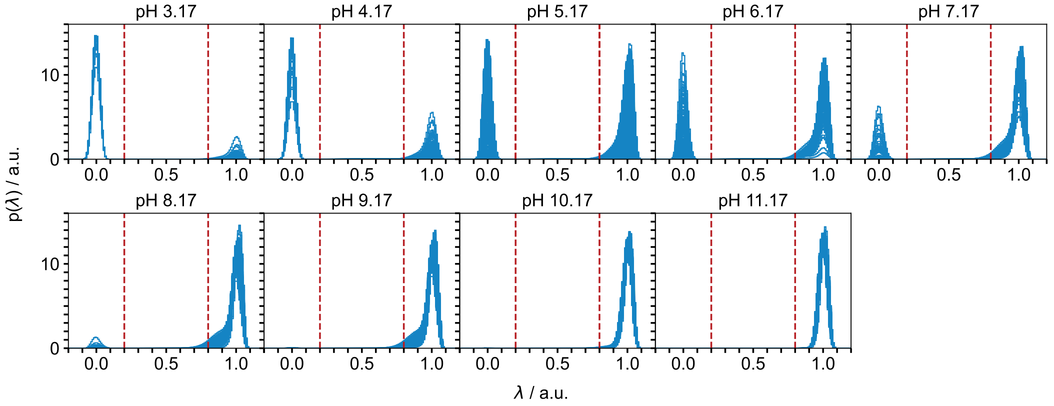

Figure S21: **λ-coordinate distributions for individual aminolipids.** Probability density distributions of the λ-coordinate are shown for simulations of D‡, and F$^{0-6\ddagger}$ (see Tab. 1). λ values within the interval $[0.2 \ldots 0.8]$, indicated by dashed vertical lines, are classified as unphysical and excluded from the remaining analysis. Histograms were computed over the full trajectories; consequently, the relative heights of the distributions do not necessarily represent fully equilibrated end states.

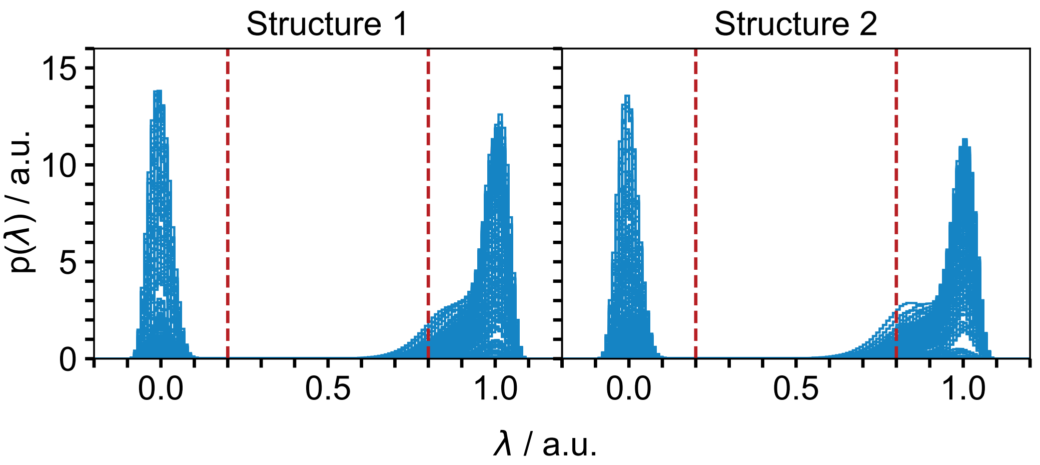

Figure S22: **λ-coordinate distributions for individual aminolipids.** Probability density distributions of the λ-coordinate are shown for simulations of systems $G^{\ddagger}$ (see Tab. 1) (LNP mixture containing mRNA). λ values within the interval $[0.2 \ldots 0.8]$, indicated by dashed vertical lines, are classified as unphysical and excluded from the remaining analysis. The mRNA-containing systems exhibit the largest deviation from the expected distribution shape, suggesting that interactions with the polynucleotides pushes the λ-coordinate toward the edges of the potential barrier from V^{bias}. Histograms were computed over the full trajectories; consequently, the relative heights of the distributions do not necessarily represent fully equilibrated end states.

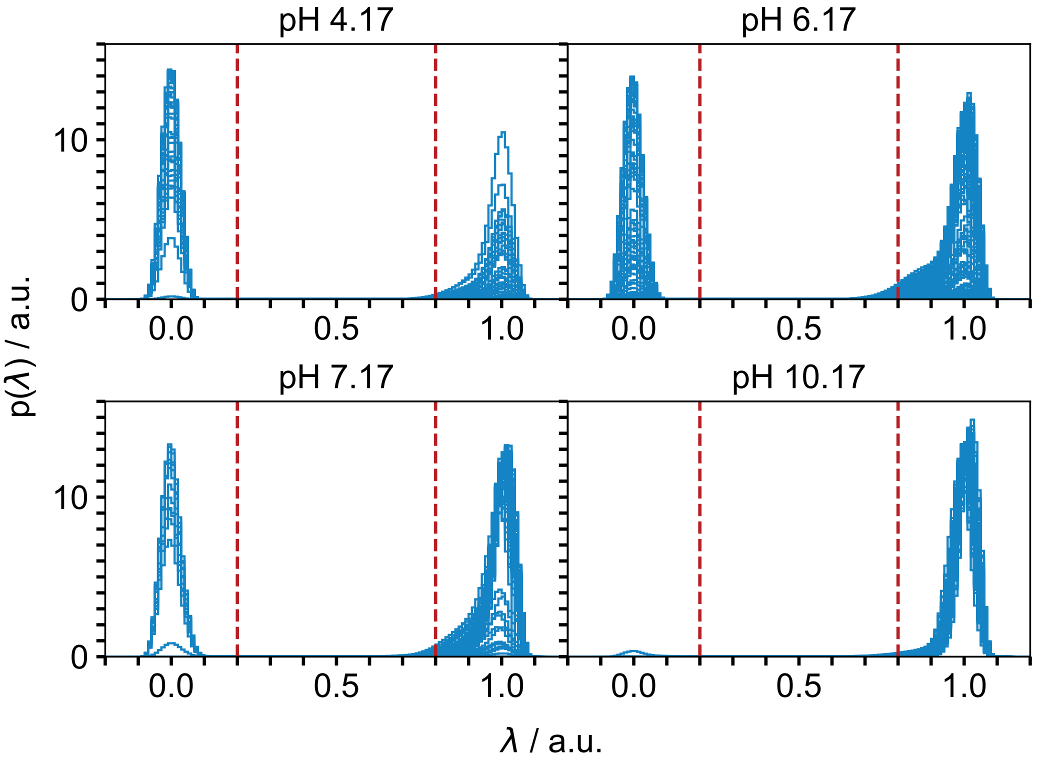

Figure S23: **λ-coordinate distributions for individual aminolipids.** Probability density distributions of the λ-coordinate are shown for simulations of systems $H^{0-3\ddagger}$ (see Tab. 1) (LNP mixture containing TNS). λ values within the interval $[0.2 \ldots 0.8]$, indicated by dashed vertical lines, are classified as unphysical and excluded from the remaining analysis. Histograms were computed over the full trajectories; consequently, the relative heights of the distributions do not necessarily represent fully equilibrated end states.

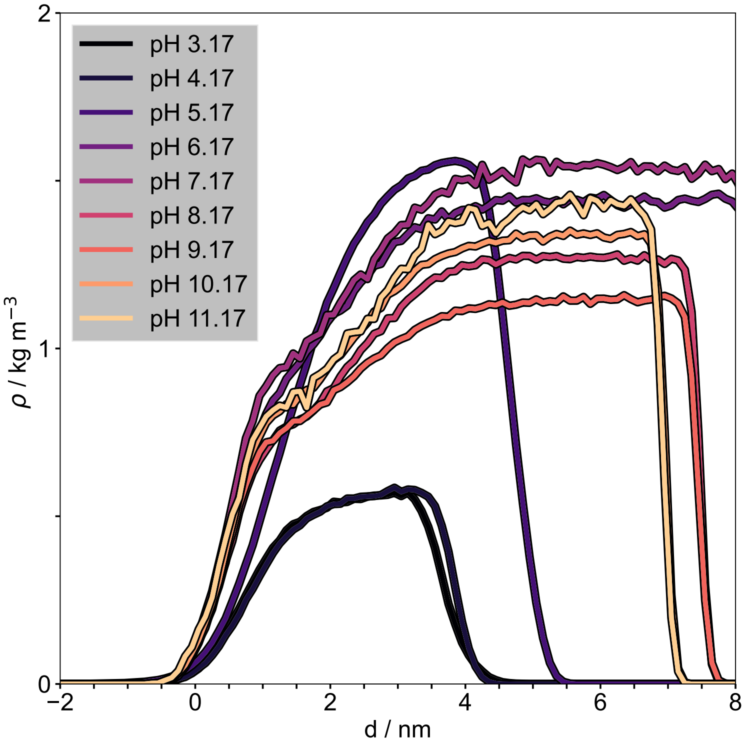

Figure S24: Average mass density profiles of the buffer particles relative to the membrane surface—defined by the median z-position of DSPC phosphorus atoms in each leaflet—were calculated for systems $D^{\ddagger}{}_{\text{pH 3.17–4.17}}$ and $F^{0-6\ddagger}$(see Tab. 1). Averages were calculated after the membrane thickness reached equilibrium; for details, see "Assessment of simulation convergence and error estimation" in the Methods section. As anticipated from the parameterization strategy of Buslaev *et al.*,[4] the buffer particles avoid the hydrophobic region of the membrane.

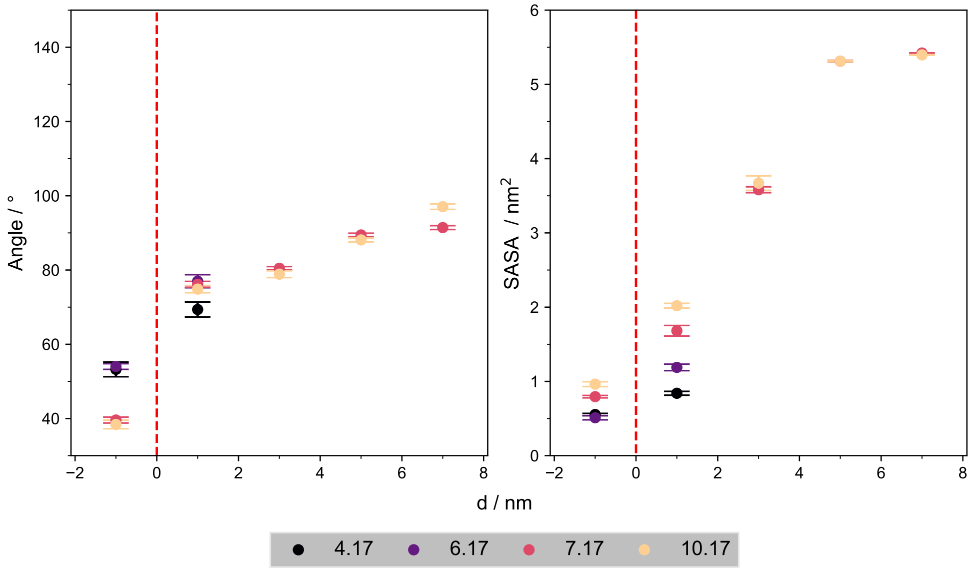

Figure S25: **a** Average angle of TNS relative to the membrane normal (i.e., the z-axis), and **b** solvent-accessible surface area (SASA) of TNS at different pH values, shown as a function of the distance from the median z-position of DSPC phosphorus atoms and averaged over both leaflets (bin width 2.0 nm). Negative bin centers correspond to TNS molecules within the membrane, while positive bin centers correspond to molecules above the membrane surface. For the angle calculation, a vector pointing from the tail group to the headgroup was defined for each TNS molecule. Error bars represent the standard error of the mean calculated via block averaging.[3] Mean values were calculated after the average SASA reached equilibrium (see "Assessment of simulation convergence and error estimation").

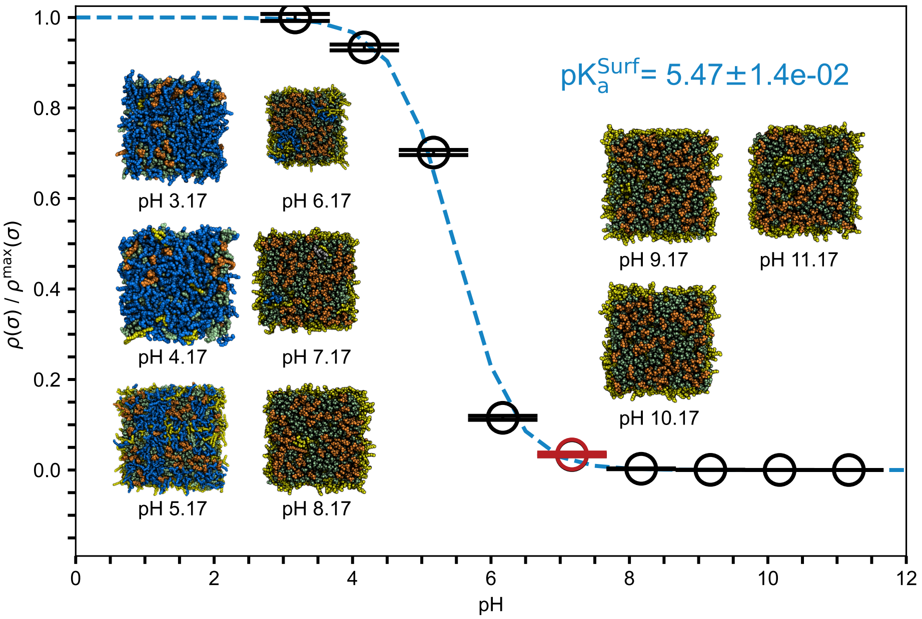

Figure S26: pH-dependent surface charge density of LNP-mimetic systems. The calculation included only protonated ALC-0315 aminolipids within 1.8 nm of, or above, the median position of the DSPC phosphorus atoms in one leaflet. The number of protonations was divided by the respective box area. Charge densities were then averaged over both leaflets in every frame. Error bars represent the standard error of the mean, calculated via block averaging.[3] The values were normalized by the maximal charge density, $\rho^{\max}(\sigma)$, derived from system D‡ at pH 3. The red marker represents the surface charge density of the LNP-mimetic system containing mRNA (Structure 1). The results indicate that the presence of mRNA inside the LNP does not affect the surface charge density at pH 7. Averages were calculated after the S^{deprot} values reached equilibrium. Errors in the fitted parameters were estimated using bootstrapping.[5] Assuming that the averaged scaled surface charge density is normally distributed at each pH value, synthetic datasets were generated by sampling from normal distributions with means and standard deviations given by the estimated means and standard errors. This procedure was repeated 100,000 times. The reported pK_a^{LNP} value with the error bar correspond to the mean and the 95% confidence interval of the resulting bootstrap distribution. Fitting was performed using iteratively reweighted least squares (see Methods). Inset images show the surface of one leaflet of the LNP-mimetic extracted from the last simulation frame: deprotonated ALC-0315 (*yellow*), protonated ALC-0315 (*blue*), DSPC (*orange*), and cholesterol (*green*). ALC-0159, and solvent are not shown. All pictures of atomistic structures were rendered with PyMOL.[6]

References

[1] G. B. Brandani, M. Schor, C. E. Macphee, H. Grubmüller, U. Zachariae, D. Marenduzzo, *PLoS One* **2013**, *8*, 6 e65617.

[2] L. Bartoš, P. Pajtinka, R. Vácha, *SoftwareX* **2025**, *31*, 102254 102254.

[3] B. Hess, *J. Chem. Phys.* **2002**, *116*, 1 209.

[4] P. Buslaev, N. Aho, A. Jansen, P. Bauer, B. Hess, G. Groenhof, *J. Chem. Theory Comput.* **2022**, *18*, 10 6134.

[5] M. F. Ergüder, M. Deserno, *J. Chem. Phys.* **2021**, *154*, 21 214103.

[6] Schrödinger, LLC, The PyMOL molecular graphics system, version 3.1, **2024**, https://github.com/schrodinger/pymol-open-source.
